# Preliminary Study of S100B and Sema3A Expression Patterns in Regenerating Muscle Implicates P75-Expressing Terminal Schwann Cells and Muscle Satellite Cells in Neuromuscular Junction Restoration

**DOI:** 10.3389/fcell.2022.874756

**Published:** 2022-07-18

**Authors:** Nasibeh Daneshvar, Judy E. Anderson

**Affiliations:** Department of Biological Sciences, University of Manitoba, Winnipeg, MB, Canada

**Keywords:** neuromuscular junctions (NMJs), neuritogenesis, myogenesis, P75 nerve growth-factor receptor, S100B, SEMA3A, principal component analysis

## Abstract

Terminal Schwann cells (TSCs) help regulate the formation, maintenance, function, and repair of neuromuscular junctions (NMJs) and axon guidance after muscle injury. Premature activation of muscle satellite cells (SCs), induced by isosorbide dinitrate (ISDN) before injury, accelerates myogenic regeneration, disrupts NMJ remodeling and maturation, decreases Sema3A protein-induced neuro-repulsion, and is accompanied by time-dependent changes in S100B protein levels. Here, to study the effects of premature SC activation on TSCs and SCs, both expressing P75 nerve growth-factor receptor, *in situ* hybridization was used to identify transcripts of S100B and Sema3A, and the number, intensity, and diameter of expression sites were analyzed. The number of sites/fields expressing S100B and Sema3A increased with regeneration time (both *p* < 0.001). Expression-site intensity (S100B) and diameter (S100B and Sema3A) decreased during regeneration (*p* = 0.005; *p* < 0.05, *p* = 0.006, respectively). P75 protein colocalized with a subset of S100B and Sema3A expression sites. Principal component analyses of gene expression, protein levels, and histological variables (fiber diameter, vascular density) in control and ISDN-pretreated groups explained 83% and 64% of the dataset variance, respectively. A very strong loading coefficient for colocalization of P75 protein with S100B and Sema3A mRNAs (0.91) in control regenerating muscle dropped markedly during regeneration disrupted by premature SC activation (−0.10 in Factor 1 to 0.55 in Factor 3). These findings strongly implicate the triple-expression profile by TSCs and/or SCs as a strong correlate of the important synchrony of muscle and nerve regeneration after muscle tissue injury. The results have the potential to focus future research on the complex interplay of TSCs and SCs in neuromuscular tissue repair and help promote effective function after traumatic muscle injury.

## Introduction

Terminal Schwann cells (TSCs) mediate axon growth during regeneration in the peripheral nervous system (Son and Thompson, 1995). TSCs, among other cells including neurons, astrocytes, and muscle satellite cells (SCs) associated with myofibers, produce S100B, a calcium-handling protein used to study the location and activity of TSCs (Reynolds and Woolf, 1992; Sorci et al., 2004; Donato et al., 2009; Donato et al., 2013). In contrast, myofibers in skeletal and cardiac muscle primarily produce S100A (Donato et al., 2013). S100B promotes myoblast proliferation and delays differentiation after muscle injury (Riuzzi et al., 2016; Riuzzi et al., 2017). S100B is a potent signaling protein and can act through extracellular and/or intracellular pathways (Donato et al., 2013). Interestingly, when S100B abnormally persists in regenerating muscle, it prolongs myoblast proliferation, promotes macrophage infiltration, and delays the transition of macrophages to the anti-inflammatory phenotype (Riuzzi et al., 2017; Sagheddu et al., 2018). Indeed, S100B is the ligand of RAGE, the receptor for advanced glycation end-products; at high levels, S100B induces muscle atrophy and other hallmarks of cancer cachexia (Chiappalupi et al., 2020). In addition, S100B levels respond to synaptic activity, nerve damage, and neuromuscular junction (NMJ) maturation and play a role in Wallerian degeneration (Donato et al., 2013).

We recently reported that the S100B protein level in muscle tissue increased when SCs were activated for 2 days before cardiotoxin (CTX) injury (Daneshvar et al., 2020). However, since the S100B level did not change after a traumatic muscle-crush (CR) injury, findings suggested that S100B signaling and TSCs are selectively involved in mediating the outcome of myogenic repair after CTX damage to fibers (Daneshvar et al., 2020). While both CR and CTX injuries induce muscle-fiber damage, they are distinguished by the accompanying presence (CR) or absence (CTX) of direct trauma to motor axons. Although NMJs are often restored at their original synaptic sites on regenerated fibers after CTX damage (McMahan et al., 1980), growing evidence suggests that new NMJs are formed by regenerated axons that reach new fibers called myotubes after traumatic muscle CR injury (Grumbles et al., 2012; Kang and Lichtman, 2013).

Both skeletal muscle and peripheral nerve tissues have distinct abilities to regenerate and restore function after injury (Darabid et al., 2014; Domingues-Faria et al., 2016). Muscle tissue regenerates stepwise after injury through SC activation (Anderson, 2000; Tajbakhsh, 2003) and proliferation and myoblast differentiation and fusion to form new fibers (Schmalbruch and Lewis, 2000; Chen et al., 2020). Subsequent maturation and remodeling follow after fibers are innervated and function imposes adaptations (Carlson and Faulkner, 1983; Chargé and Rudnicki, 2004; Anderson, 2006; Tatsumi and Allen, 2008; Egan and Zierath, 2013; Qaisar et al., 2016; Wosczyna and Rando, 2018) that are independent of further precursor fusion (Englund et al., 2020; Englund et al., 2021). Interestingly, SCs are also implicated in regulating the extension of motor neurites toward synaptic regions of regenerating fibers *via* semaphorin3A (Sema3A) secretion (Tatsumi et al., 2009; Anderson et al., 2017; Tatsumi et al., 2017), although the nature of potential SC interactions with TSCs during NMJ restoration is not known.

Recent experiments showed that myogenic repair was accelerated when SCs were activated before injury (Daneshvar et al., 2020) using isosorbide dinitrate (ISDN) to deliver nitric oxide, a mediator of early activation events (Anderson, 2000). Premature SC activation before injury markedly increased S100B protein levels after CTX injury compared to regenerating muscle in control untreated mice. The results suggested that premature activation may have uncoupled TSC–SC interactions during muscle tissue repair, since motor neurite and NMJ patterning 8–10 days after CTX injury were also disrupted by premature SC activation, as shown by the reduced colocalization of pre- and postsynaptic NMJ features. In addition, the level of Sema3A-65, a less chemorepulsive form of Sema3A, was higher in muscles regenerating after CR injury with premature SC activation compared to controls; that increase may permit the abnormally early ingrowth of neurites toward new myotubes and result in asynchronous maturation of pre- and postsynaptic regions, processes partly enabled by TSCs (Daneshvar et al., 2020).

Neurotrophic factors facilitate survival, neurite outgrowth, differentiation, and functional plasticity in several neuronal populations in the central and peripheral nervous systems. TSCs and SCs both express neurotrophin receptors, including the P75 nerve growth-factor receptor (P75). P75 is a glycoprotein (Baron et al., 1994; Mousavi and Jasmin, 2006) that functions along with tyrosine receptor kinases to mediate bidirectional crosstalk in response to neurotrophins (Yano and Chao, 2000). Nerve growth factor and P75 are both expressed in developing muscle and are upregulated during differentiation *in vitro*; such upregulation triggers changes involved in myoblast fusion into myotubes (Ip et al., 2001). P75 in muscle is also upregulated during Wallerian degeneration (Kirsch et al., 2009) and pathologies such as muscular dystrophy (Toti et al., 2003) and amyotrophic lateral sclerosis (Küst et al., 2002). Interestingly, P75 expression is mediated by the muscle regulatory factor MyoD (Seidl et al., 1998), expressed by activated muscle SCs and proliferating myoblasts shortly after activation and early in regeneration (Megeney et al., 1996; Cornelison and Wold, 1997; Cooper et al., 1999; Sabourin et al., 1999). P75 expression is therefore associated with both myogenesis and neuronal processes including survival, axon growth, and cell death (Roux and Barker, 2002; Schor, 2005; Deponti et al., 2009).

After denervation, signals released by TSCs serve as a guidance substrate for regenerating motor neurites and can induce the formation of new motor boutons at synapses (Son and Thompson, 1995). Premature SC activation disrupted the normal synchrony of myogenic regeneration and NMJ restoration (Daneshvar et al., 2020) and suggested the involvement of TSCs. This preliminary study of P75-expressing cells at NMJ regions by *in situ* hybridization (ISH; to specifically identify the source of Sema3A and S100B synthesis, rather than identifying the location of those proteins) and immunostaining (for P75 protein) was used to extend the scope of previous experiments on the same mice. By identifying the population of cells responsive to nerve growth factors and synthesizing Sema3A and S100B in a model that included treatment-induced premature SC activation, the hypothesis that synchrony of TSC and SC activation following muscle damage plays a role in myogenic regeneration and NMJ restoration was tested.

## Materials and Methods

This study is a preliminary application of ISH and principal component analysis (PCA) on tissues remaining from a previously reported experiment (Daneshvar et al., 2020).

### Tissues

Mice (10-week-old C57BL6 mice) were housed and treated according to regulations set by the Canadian Council on Animal Care, approved by the University of Manitoba (protocol F14-015). Half the mice received oral ISDN (66 mg/kg in canola oil at a volume of 2.7 μL/g body weight/day) for 2 days before muscle injury. The left tibialis anterior muscle was injured either by traumatic crush (CR) or percutaneous CTX injection (Sigma-Aldrich, Mississauga, ON, Canada), as reported for the same animals (Daneshvar et al., 2020). Animals (CTX or CR, with or without ISDN pretreatment) were euthanized immediately after injury (day 0) or 4–10 days after injury. A small number of animals contributed data to the present study (*n* = 1–4 for various time points in different runs of staining); this was due to the need to identify NMJ regions with a previously reported combination of immunostaining, histochemistry, and silver staining and other staining approaches in the earlier report (Daneshvar et al., 2020) and data collected from alkaline phosphatase staining for blood vessels as reported in other studies (Leiter et al., 2012). Consequently, the current findings are considered a preliminary characterization of P75-expressing cells during muscle regeneration. From the original 80 animals previously reported, 1-2 slides (each with 2 sections) were available for 26 mice; this included independent samples from 12 control mice and 14 mice pretreated with ISDN, spread over groups injured by CR (*n* = 18) and CTX (*n* = 8).

### Immunostaining and *In Situ* Hybridization

NMJ regions were identified in one set of slides using a combination of silver staining for intramuscular presynaptic neurites and axon terminals, histochemical staining for acetylcholinesterase (AchE) plaques, and direct fluorescent staining for acetylcholine receptors (AchRs) using alpha-bungarotoxin (BTX) (Daneshvar et al., 2020). For staining for localized S100B protein on a subset of the same slides, rabbit polyclonal anti-S100B antibody (orb228251, Biorbyt, 1:3,000), blocking, and goat anti-rabbit IgG DyLight650 (ab96902, Abcam, 1:200) were used to identify TSCs.

ISH experiments were used to detect transcripts of S100B and Sema3A expression in NMJ-containing regions of muscle sections on a second set of slides. The ISH protocol used an RNAScope V2 multiplex fluorescent detection reagent kit (ACD 323100, Advanced Cell Diagnostics, Hayward, CA) and equipment including the HybEZ oven (ACD 310010), HybEZ humidity control tray (ACD 310012), and EZ-Batch slide rack (ACD 310017), according to the manufacturer’s instructions. Sections were counterstained with 4′,6-diamidino-2-phenylindole (DAPI, Sigma, D9542, 1:10,000) to identify cell nuclei. Negative- and positive-control probes provided with the kit allowed sensitivity and specificity of the protocol to be standardized prior to conducting ISH on experimental paraformaldehyde-fixed, frozen sections, according to the manufacturer’s instructions. Sequences of target probes, preamplifier, amplifier, and label probes supplied by ACD are available through open access at http://jmd.amjpathol.org (AMP, 2020). Fluorochromes for fluorescent detection of Sema3A and S100B transcripts were from Molecular Probes (Invitrogen, Eugene, OR). Slides from different treatment groups were processed in groups of 10 on multiple runs of the 2-day ISH protocol. Some sections were lost during multiple washing steps.

ISH was combined with immunostaining for P75 expressed by SCs and TSCs for CR and CTX injury only at day 0 from control and ISDN-treated groups. After the ISH protocol, slides were washed (3 min × 2 min) with Tris-buffered saline containing 0.1% Triton-X100 and prepared for immunostaining for P75 protein. Slides were blocked with unconjugated Fab fragment goat anti-mouse IgG (115-007-003, Jackson ImmunoResearch, 1:200) and unconjugated Fab fragment goat anti-rabbit IgG (11-007-003, Jackson ImmunoResearch, 1:200). P75 protein was detected using a mouse anti-P75 NGFR primary antibody (sc-271708, Santa Cruz, 1:1,500), followed by a secondary goat anti-mouse IgG conjugated with DyLight488 (ab96879, Abcam, 1:200) and mounted in ProLong gold antifade mounting medium (P36987, ThermoFisher Scientific, Waltham, MA). For this subset of slides, the number of sites of P75 protein localization was determined.

### Imaging and Expression Analysis

NMJ regions were observed with a ×40 objective on an Olympus I × 71 epifluorescence microscope; 10 non-overlapping fields of one slide/animal were imaged using a PXL37 CCD camera (Photometrics, Tucson, AZ). Fields of damaged fibers or myotubes in NMJ regions displaying one or more expression sites for S100B (red fluorescence) and/or Sema3A (green fluorescence) transcripts, with or without P75 protein (white immunofluorescence), were photographed in all three wavelengths and in some cases, by differential interference contrast (DIC) for orientation. The arrangement of nuclei identified by DAPI counterstaining helped distinguish fibers (peripheral nuclei) from regenerating myotubes (central nuclei). Images for illustration include the use of ×40 and ×100 objectives.

Using a standardized calibration of color and intensity threshold for each channel (Fisher, 2019), the number, intensity (arbitrary units in grayscale), and maximum diameter (in pixels) were measured for each site of S100B and Sema3A mRNA using Celleste Image Analysis Software v5.0 (InVitrogen, ThermoFisher Scientific, Waltham, MA). By overlaying images of fluorescence channels, regions of colocalized expression of P75 protein (by ICC) with S100B and Sema3A transcripts (by ISH) could be outlined and measured for the area (in pixels^2^) by the software (abbreviated S + S + P75) in the 10 imaged fields.

### Statistical Analysis

Data were collected in Microsoft Excel 2016 spreadsheets, decoded, and analyzed by 3-way ANOVAs using Jamovi v1.1.9 open-access software with *post hoc* Tukey’s test of means, as appropriate, and Pearson’s linear regressions. A probability of *p* < 0.05 was used to indicate significance.

PCA by XLSTAT software (Addinsoft Inc, New York, NY) was used to reduce the dimensionality of the multivariate dataset and facilitate its graphical illustration. PCA identifies those variables that contribute most to explaining overall variability in the dataset. Those variables are grouped into a small number of new components, called factors (Fs), that maintain correlations among the original variables. Three factors—F1, F2, and F3—were identified in PCA, and loading coefficients were calculated as the strength of correlation between an individual variable and each factor. The correlation circle for PCA helps interpret the numerous correlations among a large number of variables. With each of the principal components (Fs or factors), the larger the value by which a variable “loads” onto a factor (the loading coefficient), the greater the correlation of that variable with that multivariate factor revealed by PCA. Loading coefficients of 0.5 or greater were considered to indicate strong relationships. Significant inter-variable relationships were plotted on a correlation circle in which orthogonal x, y, and *z* axes, respectively, represent the multivariate factors that best explain overall variance in decreasing importance (F1 > F2 > F3) (Navarro and Foxcroft, 2018). Vectors for each variable represent the linear relationship between that variable and the three factors (axes), according to the loading coefficients with each factor (Mukaka, 2012). Angles between vectors or between a vector and a particular factor can vary from highly acute (<45°) to highly obtuse (>135°), indicating large positive or negative correlations, respectively. Bartlett’s test for sphericity, when significant, was used to reject the null hypothesis that variables are not related.

## Results

### Localization of NMJ Regions

**FIGURE 1:**
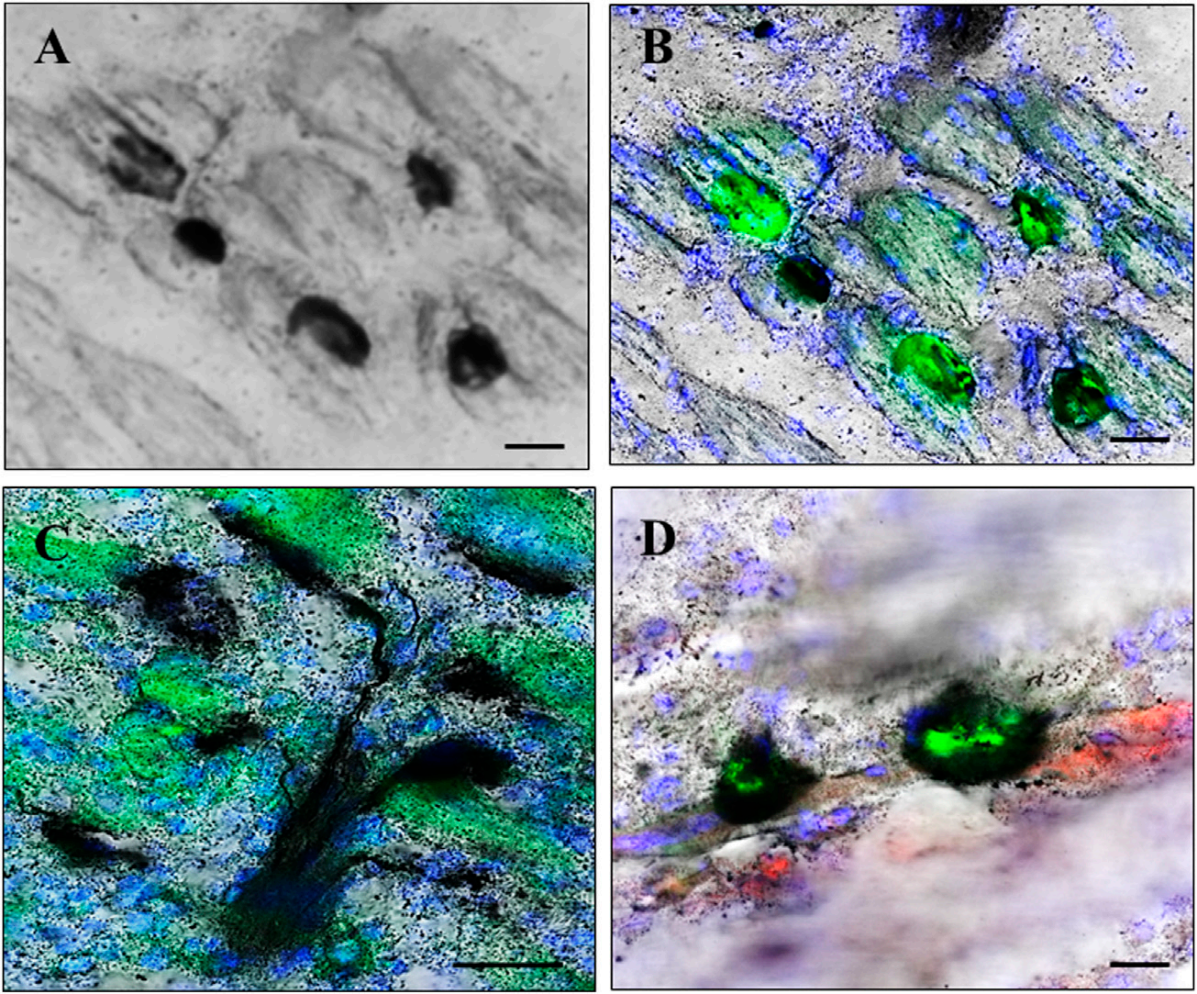
Neuromuscular junction (NMJ) regions. NMJ regions were localized in muscle using a combination of silver staining, enzyme histochemistry, direct fluorescence, and immunostaining. Presynaptic regions were identified using silver staining histochemistry for acetylcholinesterase (AchE) enzyme activity and neurites **(A)** alone, and additionally localized in combination with staining for postsynaptic acetylcholine receptor (AchR) plaques which were identified by green fluorescence after staining with FITC-conjugated α-bungarotoxin **(B–D)**. Terminal Schwann cells (TSCs) localized in some sections by further immunostaining for S100B protein, identified by red fluorescence **(D)**. **(A,B)**, and **(D)** illustrate NMJ regions in regenerating muscle on day 6 after cardiotoxin injury **(A,B)** and day 8 after muscle-crush injury **(D)**. **(C)** Illustrates presynaptic neurites branching toward regions of AchE activity closely juxtaposed to postsynaptic AchR plaques in control muscle without injury. Nuclei are counterstained blue by 4′,6-diamidino-2-phenylindole **(A,C,D)**. Bars indicate ×10 µm.

NMJ regions were identified in sections by the combination of staining for pre- and postsynaptic regions (Figure 1).

### Expression of mRNA Transcripts and Cell Localization

As the first step in understanding the potential differences in gene expression among different treatments after muscle injury, the expression level of each transcript was assessed by counting the number, overall intensity (areal density), and size (diameter) of expression sites in 10 non-overlapping 40× fields of NMJ regions. A single cell could contain one “dot” or punctum of fluorescence, ranging from a clear although modestly fluorescent spot to an intensely bright fluorescent dot. These dots are termed expression sites, where transcripts of interest are located inside cells, in the following descriptions of results per field. In addition, there was a very wide distribution of brightness as the intensity of the dots of expression ranged widely among and within sections. This variability was related to the histological region that was imaged, the number of NMJs in a particular field, as well as the architecture of NMJs and other cells in the particular field and section. The overall distribution of expression sites in a 40× field was measured as the intensity (meaning areal density) of expression.

The third parameter, the diameter of expression sites, was evaluated since the size of expression sites was observed to expression sites range from small puncta to groups, to much larger areas that could fill entire cells. The size of an expression site is related to the extent of expression by a cell population in a tissue, and the size range can show the evenness (homogeneity vs. heterogeneity) of the level of expression activity by that population. DAPI staining of nuclei helped identify the general density of individual cells and often their alignment or collection (e.g., in myotubes or at NMJ regions) and helped confirm that fibers were regenerated myotubes as they had central nuclei.

**FIGURE 2:**
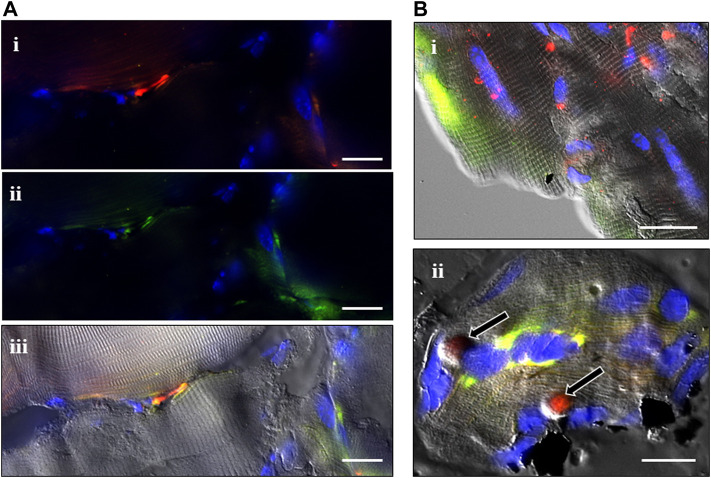
Combined *in situ* hybridization (ISH) experiments and P75 immunostaining. A combination of fluorescent ISH and immunostaining was used to localize the expression of Sema3A (green) and S100B (red) mRNAs and P75 protein (white) in the tibialis anterior muscle at different time points after cardiotoxin (CTX) and muscle-crush (CR) injury, with or without ISDN pretreatment **(A)**. CTX-injured muscle at day 0 (immediately following CTX injection and after 2 days of ISDN pretreatment) showing S100B expression (i, red fluorescence), Sema3A expression (ii, green fluorescence), and S100B and Sema3A mRNA signals merged with the differential interference contrast (DIC) image (iii) **(B)**. Images of ISH signals overlain with corresponding DIC images of the same representative fields of control untreated, uninjured muscles (i, ii) showing S100B (red) and Sema3A (green) ISH signals alone (i) or in combination with P75 immunostaining (white) (ii). **(Bii)** shows two probable terminal Schwann cells (TSCs) identified at rounded S100B-expressing regions surrounded by P75 protein (arrows). Bars indicate 10 µm **(A,Bi)** or 5 µm **(Bii)**.

**FIGURE 3:**
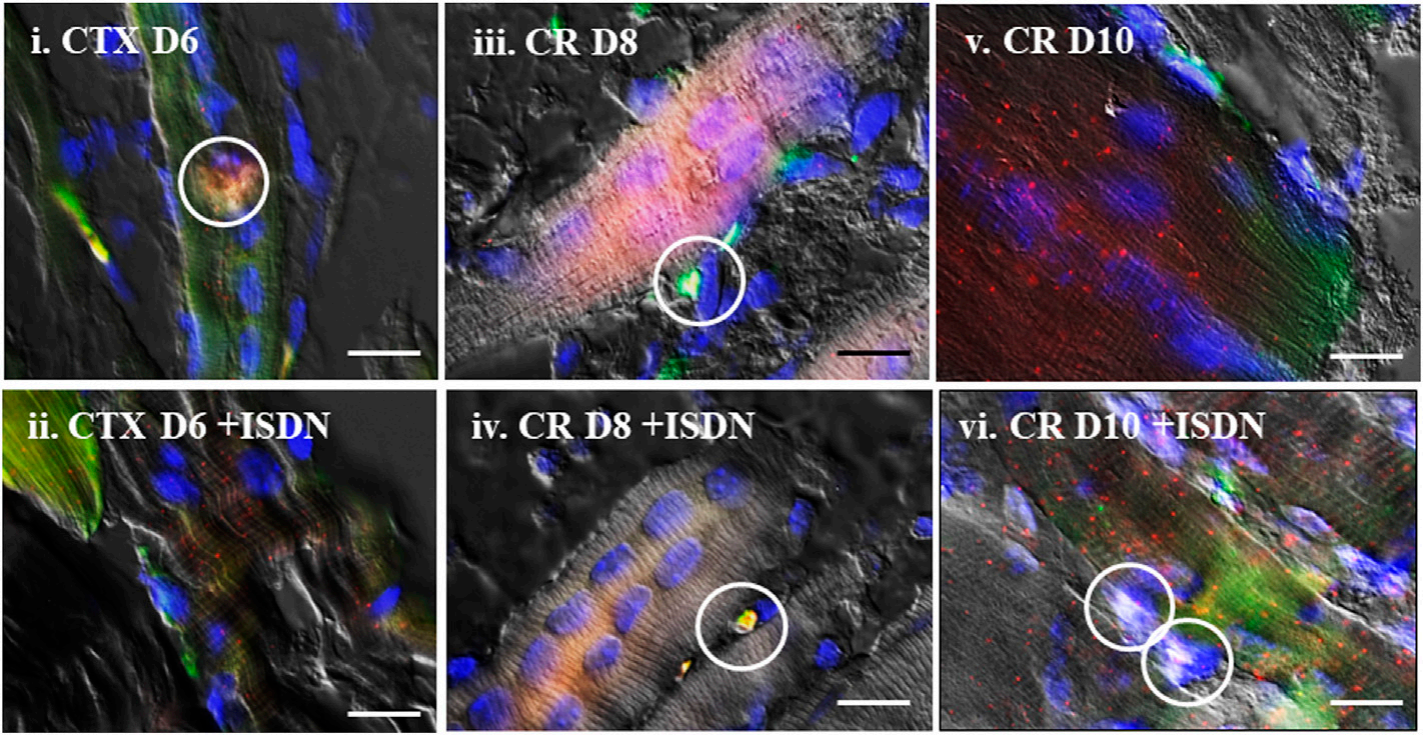
Colocalization of expression. Representative *in situ* hybridization (ISH) images merged with differential interference contrast (DIC) of the same field, showing Sema3A (green) and S100B (red) mRNA expression after cardiotoxin injury (day 6 (D6)) **(i,ii)** and after muscle-crush injury (D8, **iii,iv,** and D10, **v,vi**) without pretreatment **(i,iii,v)** and with 2 days of ISDN pretreatment **(ii,iv,vi)**. Areas of Sema3A (green) and S100B (red) mRNA expression colocalized with P75 protein (white fluorescence) in terminal Schwann cells are indicated by white circles. Nuclei are counterstained with 4′,6-diamidino-2-phenylindole (blue) in all fields. Bars indicate 10 µm.

ISH staining was examined in those NMJ-containing regions imaged from a second set of sections from the same muscles. ISH signals ranged from modest to intense fluorescence at puncta; the number of sites varied from sparse to many at each expression site, depending on treatment group, the proximity of NMJs, muscle architecture, the plane of section through the muscle and NMJ, and also the state of damage and regeneration within that region of muscle (Figure 2). Transcript expression ranged from single or collected puncta to larger-diameter sites that occasionally seemed to fill entire cells. Sites of colocalized expression for Sema3A and S100B transcripts and P75 protein identified SCs and TSCs collectively but did not distinguish between the two cell types (Figure 3). Sites of colocalized transcripts and P75 protein (S + S + P) were more prominent in CTX-injured muscles than in CR-injured muscles at a comparable time after injury regardless of treatment. Subjectively, those sites also seemed to be more frequent with ongoing regeneration and to be lower in ISDN-treated than in control groups. This was the approach utilized to identify the expression of P75-positive cells expressing Sema3A and S100B transcripts.

### *In situ* Hybridization Combined With Immunohistochemistry

**FIGURE 4:**
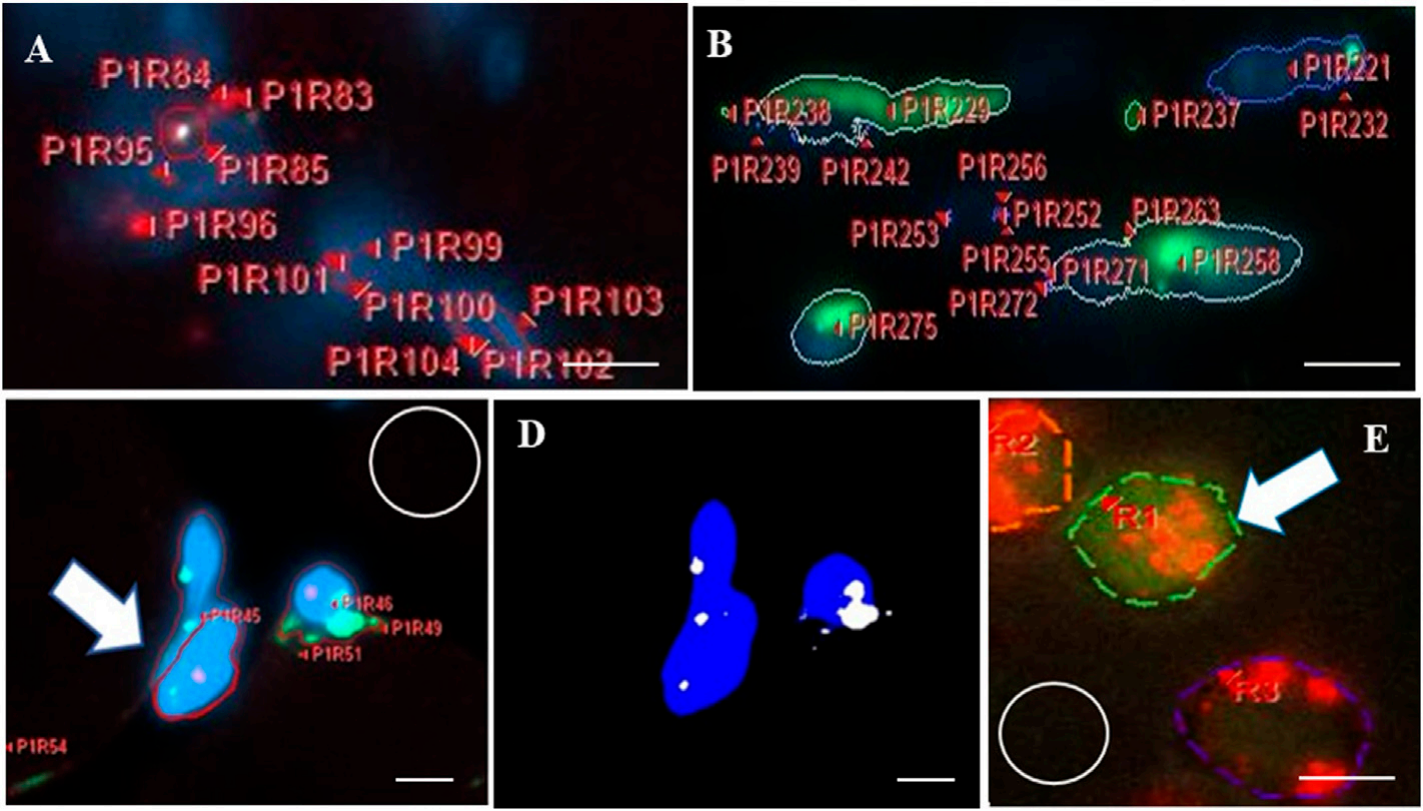
Analysis of *in situ* hybridization (ISH) signals and areas of colocalization using Celleste software. After imaging and software calibrations for brightness and magnification, fluorescent regions in each image field were viewed. The program automatically detected, color-outlined, coded, and labeled each fluorescent site with a unique alpha-numeric identifier, before counting sites and measuring each for intensity and maximum diameter. **(A,B)** show two representative highly magnified fields of muscle to illustrate the labeling and counting feature of the software applied to a single fluorochrome, both at day 0 after muscle-crush injury. Fields display numerous sites outlined for red fluorescence **(A)**, detecting S100B (outlined by red lines), or green fluorescence **(B)**, detecting Sema3A (outlined by green lines), with alpha-numeric labels. Fluorescent sites varied from tiny to large. Each irregularly shaped region of fluorescence is counted as a single expression site, according to specifications of the RNAScope v2 multiplex technology. The intensity (areal density) of each region was measured (not including non-fluorescent pixels inside an outline). The maximum diameter of each fluorescent region was measured in pixels, and data for each site in every imaged field (10 per section) were recorded and compiled in a spreadsheet. **(C)** Shows three regions of blue fluorescence (a test area of prominent 4′,6-diamidino-2-phenylindole-labeled nuclei) that are outlined in red (one is indicated by the white arrow). Overlapping areas of green fluorescence were identified using the “smart segmentation” tool. **(D)** Shows the application of the Celleste “colocalization” feature used to identify and measure the spatial overlap (area) between two (or more) different fluorescent color labels. In this example, the colocalization feature identified regions of overlap between blue and green in panel **(C)** and indicates those as the white regions shown in **(D)**. Regions of colocalization were subsequently counted by the software. **(E)** Shows a small highly magnified area from a field imaged for ISH signals in a representative muscle section showing three sites that were outlined (dashed red lines) for red fluorescence. One of those sites (white arrow) overlaps and is surrounded by green fluorescence from a second probe (dashed green outline). White circles in **(C,E)** indicate regions used to measure the background intensity. In each field, background intensity was subtracted from intensity measurements of fluorescence. Bars indicate 1.8 µm **(A,B,E)** or 3 µm **(C,D)**.

Photographs of fluorescent mRNA expression sites were analyzed in detail, at 10 NMJ-containing fields and analyzed by Celleste software tools (Figure 4) to compile information on the number of expression sites, and the intensity (areal density) of fluorescence, and the diameter of mRNA or P75 protein expression sites for each fluorochrome. The colocalization tool was used to identify and measure the spatial overlap among Sema3A and S100B transcripts and P75 immunofluorescence, to represent the cytoplasm of P75-positive cells, namely TSCs and SCs, in those regions. Table 1 shows data for measurements of the number, intensity, and diameter of expression sites from available samples.

**TABLE 1:** Expression analysis of S100B and Sema3A transcripts and P75 protein in regenerating muscle. Muscle-crush (CR)- and cardiotoxin (CTX)-injured muscles in mice without treatment (control) or after 2 days of pretreatment with isosorbide dinitrate (ISDN) from 0 to 10 days after injury (Day). Celleste software measured the expression-site number, the intensity of fluorescence (areal density, arbitrary grayscale units), and the diameter (pixels) against calibration standards. Sample size (*n*) for each entry appears in brackets; “-” indicates missing data.

	Injury	Treatment	Day	Number (*n*)	Intensity (X1,000)	Diameter
S100B mRNA	CR	control	0	4.3 ± 0.3 (3)	106.3 ± 51.1 (3)	388 ± 248 (3)
		ISDN		3.1 ± 0.1 (3)	-	403 (1)
		control	4	-	-	-
		ISDN		-	-	863 ± 66 (2)
		control	6	-	-	42 (1)
		ISDN		-	-	80 ± 16 (3)
		control	8	16.7 ± 5.5 (2)	21.8 ± 5.3 (3)	120 ± 44 (2)
		ISDN		15.2 ± 3.5 (3)	30.4 ± 13.1 (3)	-
		control	10	35.3 ± 3.1 (3)	6.0 (1)	108 ± 9.4 (2)
		ISDN		35.6 ± 7.2 (3)	12.3 ± 2.8 (4)	113 ± 16 (4)
	CTX	control	0	4.4 ± 0.1 (4)	362.3 (1)	666 ± 1.0 (2)
		ISDN		3.4 ± 1.6 (3)	-	-
		control	4	-	-	-
		ISDN		7.4 ± 0.2 (2)	-	-
		control	6	2.0 (1)	43.0 ± 35.8 (2)	266 ± 216 (2)
		ISDN		17.7 ± 10.5 (2)	121.3 ± 85.0 (2)	278 ± 66 (3)
Sema3A mRNA	CR	control	0	3.6 ± 0.2 (4)	168.4 ± 59.0 (3)	1,058 ± 74 (3)
		ISDN		2.8 ± 0.1 (3)	-	711 (1)
		control	4			-
		ISDN				1,079 ± 397 (2)
		control	6			133 (3)
		ISDN				633 ± 287 (1)
		control	8	8.2 ± 1.2 (2)	178.2 ± 54.0 (2)	1,116 ± 123 (2)
		ISDN		4.5 ± 2.1 (3)	99.4 ± 57.8 (3)	-
		control	10	11.1 ± 0.8 (2)	25.4 (1)	168 ± 28 (2)
		ISDN		8.9 ± 1.7 (4)	37.5 ± 21.1 (4)	224 ± 47 (4)
	CTX	control	0	3.7 ± 0.3 (4)	353.3 (1)	1,023 ± 25 (2)
		ISDN		4.9 ± 0.6 (3)	-	1,069 (1)
		control	4	-	-	-
		ISDN		6.2 ± 0.9 (2)	-	-
		control	6	1.9 (1)	339.5 ± 27.9 (2)	378 ± 26 (2)
		ISDN		4.6 ± 2.6 (2)	267.1 ± 83.5 (2)	1,227 ± 316 (3)
P75 protein	CR	control	0	2.8 ± 0.3 (2)	-	-
		ISDN		2.0 ± 0.3 (2)	-	-
		control	8	5.8 (1)	-	-
		ISDN		-	-	-
		control	10	-	-	-
		ISDN		-	-	-
	CTX	control	0	2.9 ± 0.7 (2)	-	-
		ISDN		2.6 ± 0.2 (2)	-	-
		control	4	-	-	-
		ISDN		5.3 (1)	-	-
		control	6	1.9 (1)	-	-
		ISDN		-	-	-

The number of S100B expression sites/field varied among groups (ANOVA F = 7.8, df = 10, *p* < 0.001) and increased significantly with regeneration time (F = 14.4, df = 4, *p* < 0.001). In controls with CR injury, the number of S100B expression sites increased from day 8 to day 10 after CR injury (*t*-test, *p* < 0.05). The number of Sema3A expression sites/field also varied among groups (ANOVA F = 4.36, df = 10, *p* = 0.002) and increased with regeneration time (F = 8.27, df = 4, *p* < 0.001). The number of S100B expression sites correlated with the number of Sema3A expression sites (*R* ^2^ = 0.721, *p* < 0.001, *n* = 26).

The intensity (areal density) of S100B expression sites decreased with regeneration time (ANOVA F = 7.95, df = 3, *p* = 0.005). The intensity of Sema3A expression sites did not change with time, type of injury, or ISDN pretreatment.

The diameter of S100B expression sites decreased with regeneration time (ANOVA F = 3.14, df = 4, *p* < 0.05; linear regression *R* ^2^ = 0.226, *p* < 0.02, *n* = 25), in particular between day 8 and day 10 after CR injury in the ISDN-treated group (*t*-test, *p* < 0.05). The diameter of Sema3A expression sites decreased with time after injury (ANOVA F = 5.8, df = 4, *p* = 0.006; linear regression *R* ^2^ = 0.281, *p* < 0.005, *n* = 26) and tended to be lower after CR than CTX injury (ANOVA F = 3.41, df = 1, *p* = 0.08).

**FIGURE 5:**
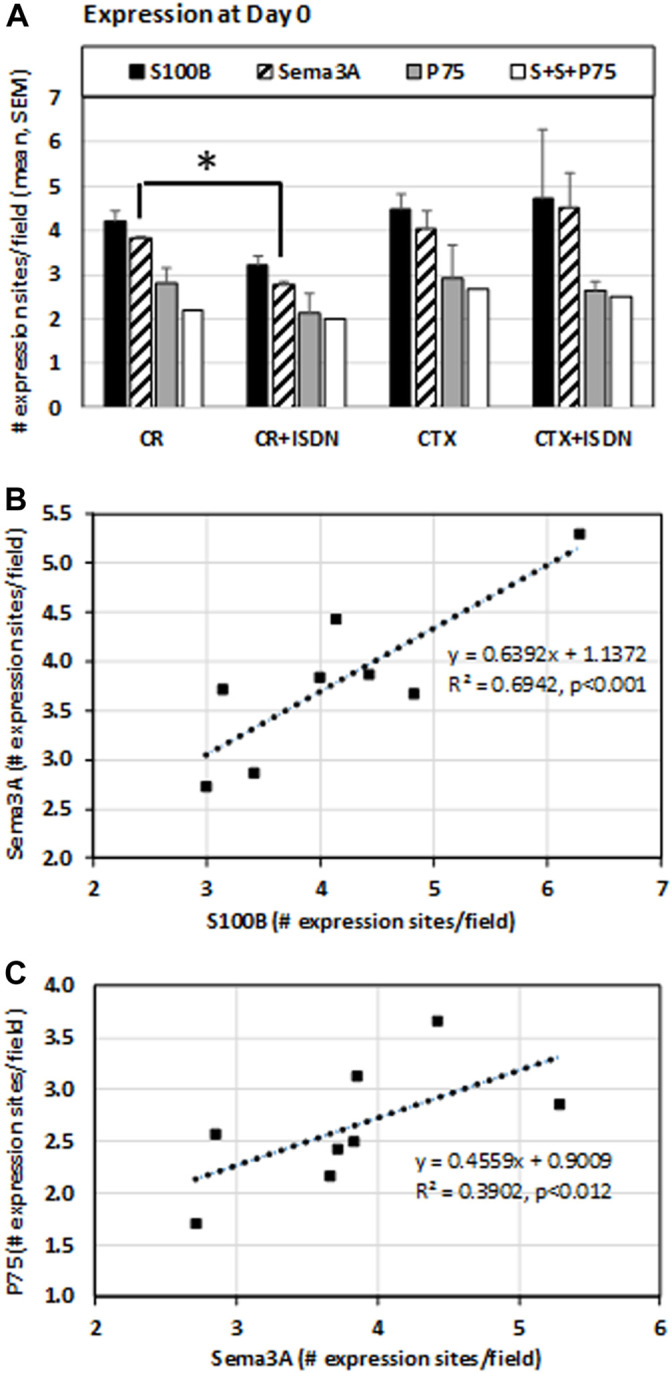
Expression at day 0 after injury. Expression sites of S100B and Sema3A transcripts and P75 protein were identified using fluorescent *in situ* hybridization and immunostaining, respectively, in muscle-crush (CR)- and cardiotoxin-injured muscles from mice either treated with ISDN for 2 days before injury (+ISDN) or controls without treatment (no ISDN). In this staining experiment, *n* = 2 per group. The number of fluorescent expression sites was identified and counted by Celleste software, which also identified the number of sites with a colocalized expression of S100, Sema3A, and P75 (indicated as S + S + P75) **(A)**. At day 0, the number of Sema3A expression sites was significantly lower after CR injury in ISDN-treated mice than in the control CR group (*p* = 0.038, *t*-test, *n* = 4) **(B)**. The number of sites expressing S100B mRNA was significantly correlated with the number of Sema3A expression sites (linear regression *R* ^2^ = 0.69, *p* < 0.001) **(C)**. The number of Sema3A expression sites was also correlated with the number of sites expressing P75 protein (linear regression *R* ^2^ = 0.39, *p* < 0.012). *p < 0.05.

The number of Sema3A expression sites was lower in CR muscles after ISDN compared with CR-control muscles (*p* = 0.038) (Figure 5A). The number of S100B expression sites in CR muscles also tended to be lower with pretreatment (*p* = −0.08). The number of Sema3A expression sites correlated with the number of S100B expression sites (*R* ^2^ = 0.69, *p* < 0.001, *n* = 8) (Figure 5B) and the number of sites stained for P75 protein (*R* ^2^ = 0.39, *p* = 0.012, *n* = 8) (Figure 5C).

The results of studying individual moieties of expression thus suggested that there were diverse changes in the expression levels, related to the number of sites, the average intensity of expression, and the diameter of expression sites for Sema3A and S100B, often with correlation among the expression of S100B and Sema3A and the number of cells that were P75-positive in the same NMJ-containing regions.

### PCA

**FIGURE 6:**
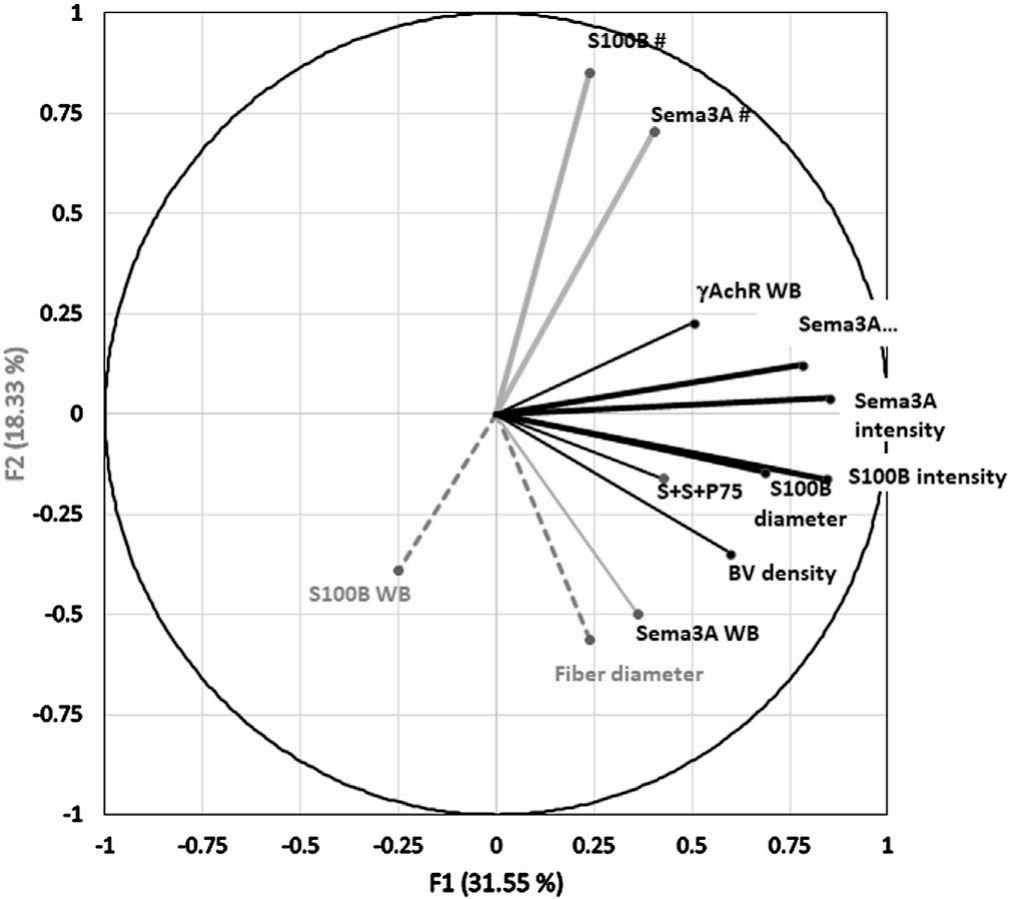
Principal component analysis (PCA) correlation circle of the overall dataset. Two factors or principal components (F1, F2) form the x and y axes of the PCA correlation circle, respectively. A third factor (F3) is on the *z*-axis, which extends upward from the x-y plane. The PCA dataset included variables from analyses of *in situ* hybridization (ISH), ISH with immunostaining (S100B and Sema3A transcripts and P75 protein), fiber diameter, and Western blotting (WB) assessments of S100B, Sema3A, and γAchR proteins from *n* = 26 mice including control and ISDN-pretreated groups after muscle-crush and cardiotoxin injury. The direction and length of the vector for each variable represent the respective loading coefficients of that variable with the three factors, F1, F2, and F3, as orthogonal axes. The endpoint position of each vector on the x-y grid represents the loading (or correlation) score of data collected for that variable (here, including all injury and treatment groups) on each of the F1 and F2 in the PCA plot. The factor to which a particular variable loads most strongly is indicated as a black line (to F1), a gray line (to F2), and gray dashes (to F3). Vectors are labeled for each variable as follows: WB for Western blotting data; BV for blood vessel density; and S + S + P75 for the number of overlapping expression sites for all three, Sema3A and S100B mRNAs and P75 protein. Data for fiber diameter, blood vessel number, and Western blotting were reported previously (Daneshvar et al., 2020). Table 2 provides the loading coefficients for each variable in this PCA with each of the three factors.

Multivariate PCA was used to study gene and protein expression (by ISH and immunostaining) in the context of the state of muscle regeneration and protein levels extant in the muscles at the time of sampling. The expression of S100B and Sema3A transcripts and P75 protein after CR and CTX injury in control and ISDN-pretreated mice was analyzed together with previously reported data for the same experiment (Daneshvar et al., 2020) on fiber diameter, blood vessel density, and protein levels of Sema3A-65, S100B, and γAchR (Figure 6). Positive correlations of Sema3A and S100B intensity and diameter loaded strongly on F1 and accounted for 31.55% of the dataset variance (Table 2). For F2, strong loading by the number of expression sites for Sema3A and S100B explained 18.33% of the dataset variance. F1 and F2 together accounted for 49.88% of the dataset variance (Bartlett’s test *p* < 0.05). The S100B protein level was highly correlated with fiber diameter, and both variables loaded strongly onto F3, explaining 13% of the overall variance (Figure 6; Table 2). Therefore, the combination of all variables for the two models of muscle injury (CR- and CTX-induced damage), and both control and ISDN-treated mice, suggested that PCA found consistent correlations among variables that explained a significant proportion of variability, almost 63%, of findings from the different treatment groups.

**TABLE 2:** Loading correlations of variables onto 3 factors identified by principal component analysis of the full dataset of muscles (*n* = 26) regenerating from injury with or without pretreatment by ISDN before injury. WB indicates protein assays by Western blotting, BV = blood vessel, “S + S + P75” indicates the number of regions in which P75 protein (by immunostaining) was colocalized with both S100B and Sema3A transcripts (by *in situ* hybridization, ISH). # indicates the number of expression sites by ISH. Loading coefficients greater than 0.5 (in bold type) were considered strong correlations.

	Factor 1	Factor 2	Factor 3
**Fiber diameter**	0.239	**−0.563**	**0.692**
**S100B WB**	−0.249	−0.389	**0.738**
**Sema3A WB**	0.362	−0.498	−0.240
**γAchR WB**	**0.505**	0.227	0.019
**BV density**	**0.598**	−0.348	−0.079
**S + S + P75**	0.429	−0.162	0.327
**Sema3A #**	0.403	**0.705**	0.475
**S100B #**	0.239	**0.850**	0.219
**S100B intensity**	**0.843**	−0.162	−0.061
**Sema3A intensity**	**0.852**	0.040	−0.129
**S100B diameter**	**0.686**	−0.145	−0.257
**Sema3A diameter**	**0.781**	0.123	0.124

Loading coefficient greater than 0.5 (in bold type) were considered strong correlations.

**FIGURE 7:**
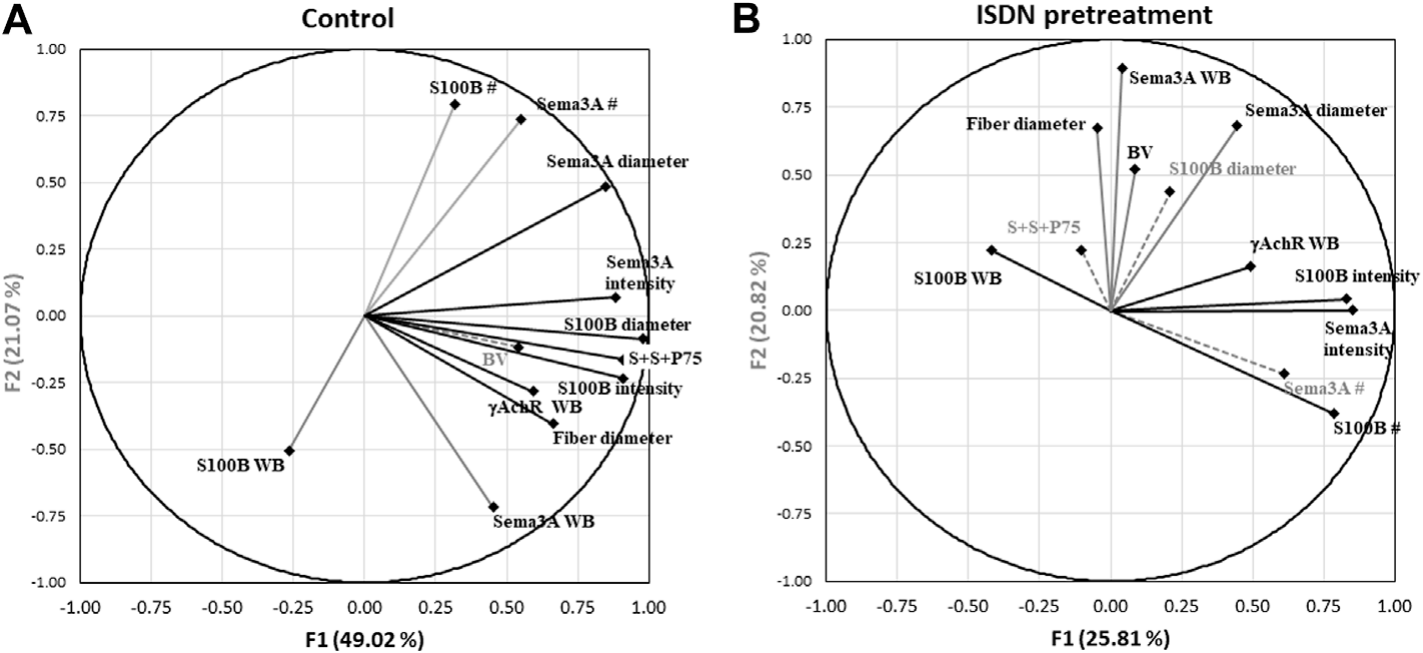
Principal component analysis (PCA) correlation circles of control and ISDN-treated groups of regenerating muscle. Factors F1 and F2 form the x and y axes of each PCA correlation circle, respectively. A third factor (F3) is on the *z*-axis, which extends upward from the x-y plane. PCA datasets included variables from analyses of *in situ* hybridization (ISH), ISH with immunostaining (S100B and Sema3A transcripts and P75 protein), fiber diameter, and Western blotting (WB) assessments of S100B, Sema3A and γAchR proteins from both muscle-crush (CR)- and cardiotoxin (CTX)-injured groups. The direction and length of the vector for each variable represent the respective loading coefficients of that variable with the three factors, F1, F2, and F3, as orthogonal axes. The endpoint position of each vector on the x-y grid represents the loading (or correlation) score of data collected for that variable (here, including all injury and treatment groups) on each of the F1 and F2 in the PCA plot. The factor to which a particular variable loads most strongly is indicated as a black line (to F1), a gray line (to F2), and gray dashes (to F3). **(A)** Correlation circle for untreated control muscles regenerating from CR and CTX injury (*n* = 12). **(B)** Correlation circle for muscles regenerating from CR and CTX injury after 2 days of pretreatment with ISDN (*n* = 14). Vectors are labeled for each variable as follows: WB for Western blotting data; BV for blood vessel density; and S + S + P75 for the number of overlapping expression sites for all three, Sema3A and S100B mRNAs and P75 protein. Data for fiber diameter, blood vessel number, and Western blotting were reported previously (Daneshvar et al., 2020). Tables 3 and 4 provide the loading coefficients of each variable in the correlation circles shown in **(A,B)**, respectively.

Two further PCAs were run to separate the multivariate relationships of untreated control and ISDN-pretreated groups of regenerating muscle. In controls (Figure 7A; Table 3), combined P75, S100B, and Sema3A expression, fiber diameter, and the intensity and diameter of S100B and Sema3A expression sites were highly loaded on F1, representing 49% of the variance. Protein levels (S100B and Sema3A), the number of expression sites (S100B and Sema3A), and fiber diameter were loaded on F2 and explained 21% of the variance. F3 explained a further 13% of the variance and included blood vessel density and S100B protein level. In muscles regenerating after the ISDN pretreatment that had disrupted NMJ restoration and accelerated myogenic repair, the PCA plot contrasted markedly from the PCA circle for untreated controls. Very different variables contributed to F1 (intensity of S100B expression, number of expression sites for S100B and Sema3A, and γAchR protein level) and explained only 26% of the variance. F2 comprised Sema3A protein level, fiber diameter, blood vessel density, and diameter of Sema3A expression site and explained 21% of the variance. F3 was composed of variables for fiber diameter, the number of Sema3A expression sites, and the number of sites that colocalized Sema3A and S100B transcripts and P75 protein and explained 17.7% of the overall variance (Figure 7B; Table 4). Thus, PCA plots highlighted the overt difference between controls and ISDN-pretreated regenerating muscles; in particular, PCA plots highlighted that more variance is explained during regeneration in control muscle than in ISDN-treated muscle with premature SC activation (83% vs. 64%), and PCA for regenerating muscles with disrupted synchrony of muscle regeneration and NMJ restoration identified colocalized Sema3A and S100B transcripts and P75 protein as an important factor.

**TABLE 3:** Loading correlations of variables onto 3 factors identified by principal component analysis of the dataset of untreated muscles regenerating from injury. WB indicates data originated from Western blotting assays, BV = blood vessel; # indicates the number of expression sites (by *in situ* hybridization, ISH); “S + S + P75” indicates the number of regions in which P75 protein (by immunostaining) was colocalized with S100B and Sema3A transcripts (by ISH). Loading coefficients greater than 0.5 (in bold type) were considered strong correlations.

	Factor 1	Factor 2	Factor 3
**Fiber diameter**	**0.662**	−0.403	−0.268
**S100B WB**	−0.266	**−0.505**	−0.498
**Sema3A WB**	0.452	**−0.717**	−0.358
**γAchR WB**	**0.593**	−0.283	−0.406
**BV density**	**0.542**	−0.116	**0.731**
**S + S + P75**	**0.910**	−0.163	0.300
**Sema3A #**	**0.552**	**0.738**	−0.285
**S100B #**	0.318	**0.792**	−0.433
**S100B intensity**	**0.908**	−0.235	−0.080
**Sema3A intensity**	**0.885**	0.070	0.207
**S100B diameter**	**0.981**	−0.087	0.086
**Sema3A diameter**	**0.850**	0.486	-0.144

Loading coefficient greater than 0.5 (in bold type) were considered strong correlations.

**TABLE 4:** Loading correlations of variables onto 3 factors identified by principal component analysis of the dataset from muscles regenerating after pretreatment with ISDN before muscle-crush or cardiotoxin injury. WB indicates protein assays by Western blotting, BV = blood vessel; # indicates the number of expression sites (by *in situ* hybridization, ISH); “S + S + P75” indicates the number of regions in which P75 protein (by immunostaining) was colocalized with S100B and Sema3A transcripts (by ISH); intensity and diameter refer to measurements of fluorescent regions of S100B or Sema3A mRNA expression (by ISH). Loading coefficients greater than 0.5 (in bold type) were considered strong correlations.

	Factor 1	Factor 2	Factor 3
**Fiber diameter**	−0.048	**0.670**	**0.572**
**S100B WB**	−0.418	0.221	0.399
**Sema3A WB**	0.041	**0.891**	0.312
**Gamma WB**	0.491	0.161	0.320
**BV density**	0.086	**0.522**	−0.405
**S + S + P75**	−0.102	0.222	**0.552**
**Sema3A #**	**0.613**	−0.234	**0.651**
**S100B #**	**0.787**	−0.382	0.394
**S100B intensity**	**0.832**	0.041	−0.237
**Sema3A intensity**	**0.853**	0.000	−0.200
**S100B diameter**	0.209	0.437	−0.458
**Sema3A diameter**	0.447	**0.682**	−0.293

Loading coefficient greater than 0.5 (in bold type) were considered strong correlations.

## Discussion

The histopathological study of muscle tissue in this experiment showed that ISDN-induced premature SC activation before CR or CTX injury accelerated myogenic regeneration while disrupting and delaying the restoration of NMJs. In that study, changes in the S100B protein level implicated TSCs in the perturbation. The current study of the expression of S100B and Sema3A transcripts and P75 protein (by combined ISH/ICC), although preliminary and limited in sample size, quantified and analyzed expression in the broader context of that histopathology. PCA showed that colocalized Sema3A, S100B, and P75, suggesting the involvement of P75-NGFR signaling, is a strong correlate of the disrupted synchrony of myogenic and neurogenic regeneration produced by ISDN-induced premature SC activation prior to muscle damage.

Two-color fluorescent ISH signals were resilient to later immunostaining for P75 protein, and DAPI staining identified the nuclear position in fibers and new myotubes. Comparator slides stained for presynaptic neurites, AchE plaques, and postsynaptic AchRs provided orientation for imaging the pre- and postsynaptic areas of NMJ-containing regions. Samples were analyzed on a cell-by-cell level through histopathology and the expression of transcripts and proteins.

Features of ISH expression sites for S100B and Sema3A (number, intensity, and diameter) changed primarily with regeneration time, with a statistical trend toward significant interaction with the type of injury. The preliminary nature of this study, with a small sample size and an incomplete set of groups, is due to the use of slides remaining after the original larger study (Daneshvar et al., 2020). Nonetheless, these findings offer some insight into the nature of gene expression under different conditions: for both S100B and Sema3A transcripts, the number of expression sites increased with regeneration time while those expression sites decreased in diameter. S100B expression sites also had lower intensity as regeneration proceeded. These findings reflect that the overall gene expression level is regulated by changes in the number of transcripts and the extent of individual transcripts within cells of interest.

The overlay of S100B and Sema3A expression with P75 protein identified a population of cells receptive to NGF. TSCs were identified as sites with a colocalized expression of S100B and Sema3A transcripts plus P75 protein, while cells expressing S100B and Sema3A without P75 were considered to be non-TSCs in the presynaptic area. Nearly all P75-expressing cells also expressed both Sema3A and S100B. Since both muscle SCs and TSCs express P75, further details of their intercellular interactions await the separation of the two cell types at NMJ-containing regions. However, current findings on P75 protein colocalization with S100B and Sema3A transcripts, although only available for day 0 (immediately after CR or CTX injury), indicate that one or both cell types were present at NMJs on damaged fibers and regenerating myotubes. In addition, the extent of colocalization of S100B and Sema3A transcripts and P75, collectively representing TSCs and SCs, very probably had a major impact on the outcome of repair in muscle and NMJs, since the colocalization variable (S + S + P75) loaded very strongly on F1 (a correlation of 0.93) in control muscle and only loaded strongly onto F3 (a correlation of 0.55) in muscle with SCs in the prematurely activated state. That PCA variables explained much more of the data variability in regenerating muscle in control mice than in ISDN-pretreated mice (83% vs. 64%) further suggests that disrupted regeneration is related to changes in the activity of TSCs and SCs and their interactions, imposed by premature SC activation. This highlights the importance of synchronized processes during the effective regeneration of muscle tissue.

The application of PCA allowed further exploration of the relationships between gene expression and previously reported outcome measures for the same regenerating muscles. Data on fiber diameter, vascular density, and Sema3A, S100B, and γAchR protein levels from the previous report plus ISH and colocalization data from the current study were input together. In the overall dataset including both control and ISDN-treated groups of regenerating muscles (Figure 6; Table 2), PCA showed that the intensity and diameter of S100B and Sema3A expression sites as well as the γAchR protein level and vascular density within the muscle were strongly represented as one factor. The number of expression sites and S100B and Sema3A protein levels formed a second factor, while fiber diameter loaded onto a third factor. These findings are challenging to interpret, likely because both pretreated and control muscles were included in the analysis, given the differences between this overall PCA and the two PCAs that analyzed separate datasets for control and pretreated groups.

Probing expression separately for control and ISDN-pretreated groups showed strong support for the idea that P75-expressing cells, identified by colocalization of S100B, Sema3A, and P75, are exceedingly important during muscle tissue regeneration under normal control conditions (Figure 7A; Table 3). This was indicated by the very strong loading coefficient on F1 (0.910) for the colocalization parameter and suggests a potent interplay between TSCs and SCs, their production of S100B, Sema3A, and P75, and new fiber growth. By comparison, the loading coefficient on S + S + P75 was highly negative and much lower (−0.102) in PCA for muscles regenerating after ISDN pretreatment. This marked shift in loading for the variable representing TSCs and SCs together is consistent with a major disruption of the interplay among P75-expressing cells, NMJ restoration (since γAchR protein level is an index of denervation), and myotube growth, given that fiber diameter (along with vascular density and Sema3A protein level and expression-site diameter) loaded most strongly on factor 2 rather than on factor 1. In concert with the previous report stating that premature SC activation delayed and disrupted NMJ restoration during early regeneration (Daneshvar et al., 2020), the present result strongly suggests the need for a synchronized response by SCs and TSCs during NMJ restoration. In muscles regenerating after premature SC activation by ISDN, there was strong loading of S100B and Sema3A expression (number of sites) and S100B and Sema3A diameter and intensity of expression sites and modestly negative loading of S100B protein level (Figure 7B; Table 4). These findings, also noted for PCA of the overall dataset, suggest that premature SC activation alters post-transcriptional and post-translational regulation of S100B and Sema3A expression. The clear angular shift in vectors for each parameter between control and ISDN-treated muscles (comparing PCA circles in 7A vs. 7B) showed that the overall impact of treatment was substantial and illustrates the need to explore P75-expressing cells in skeletal muscle for their individual and combined roles in synapse maintenance and repair during regeneration. Future research to understand the individual and collective contributions of fiber, neurite, and NMJ damage to the regenerative process has the potential to reveal new rehabilitation interventions to promote fiber reinnervation after nerve injury in muscle tissue.

PCA compiled data from many assays of histological variables and Western blotting, P75 protein, and S100B and Sema3A expression to provide a broad context to processes related to P75 expression during NMJ restoration after muscle injury. For example, in control muscles, the number of sites expressing P75 protein and transcripts for Sema3A and S100B increased during regeneration from CR injury. The concurrent increase in S100B expression sites and decrease in Sema3A expression-site diameter, Sema3A-65 protein level, and myotube diameter in control CR muscles after 10 days of regeneration suggested that Wallerian degeneration, mediated by Schwann cells and macrophages, had ended by that time (Daneshvar et al., 2020) and that axon regeneration would then proceed (Menorca et al., 2013; Darabid et al., 2014; Kang et al., 2014).

Sema3A and S100B expression sites correlated with regeneration time. NMJ remodeling is accompanied by increased Sema3A expression in TSCs of type IIb/x fibers that maintain nerve-terminal growth and confine their plasticity by releasing a chemorepulsive protein (Pasterkamp and Verhaagen, 2006). Thus, the current findings suggest that the increase in Sema3A transcripts originates in TSCs. Coordinated synaptic maturation is known to rely on the interplay of synaptogenic factors released from nerve terminals, TSCs, and muscle fibers (Darabid et al., 2014), and Schwann cells are thought to promote postsynaptic differentiation and AchR transcription by expressing neuregulin-2 (Sugiura and Lin, 2011). To distinguish between SCs and TSCs, further colocalization with Pax7 mRNA or protein will be required since both express P75, which means that the relative contribution of TSCs (Darabid et al., 2014) and/or SCs (Tatsumi et al., 2009; Tatsumi et al., 2017) to the regulation of growth-cone extension by neurites in synaptic maturation that occurs during regeneration remains to be determined at the cellular level.

Although statistically derived factors identified through PCA studies are difficult to interpret due to their multivariate nature and the number of samples in this preliminary study was limited, the large explanation of variance by PCA factors in controlling regenerating muscles suggests new possibilities for the role of P75-expressing cells. Schwann cells are known to respond to axonal injury by transiently expressing several developmental genes (Lemke and Chao, 1988; Saika et al., 1991; Lee et al., 2009). P75 upregulation by mature sensory neurons after axonal injury (Hassan et al., 1994) or distal nerve stumps after nerve transection (Toma et al., 1992) was proposed as a time-dependent indicator of the injury response by TSCs and Schwann cells (Roberson et al., 1995). It would be fascinating to find that SCs show a parallel response in their expression of P75 during regeneration and synaptic restoration.

A wealth of genetic and molecular studies describe the niche environments of stem and precursor cells during muscle regeneration (Feige et al., 2018). Recent literature using cellular tracking shows that TSCs, which extend their processes after myofiber denervation and guide the growth of sprouting axons during fiber reinnervation, have a key role in maintaining and remodeling NMJs (Love and Thompson, 1998; Sanes and Lichtman, 1999; Kang et al., 2000; Reddy et al., 2003; Panzer et al., 2006). Research to identify whether Sema3A expression by SCs and/or TSCs is essential for the effective restoration of pre- and postsynaptic NMJ compartments (Buller et al., 1960; Leiter et al., 2012) will further advance our understanding of regenerative processes that promote effective function (Suzuki et al., 2013; Mizunoya et al., 2017; Tatsumi et al., 2017).

## Data Availability

The original contributions presented in the study are included in the article/Supplementary Material, further inquiries can be directed to the corresponding author.

## References

[B1] AMP (2020). The Journal of Molecular Diagnostics. America: Asip. Available from: https://jmd.amjpathol.org/.

[B2] Anderson J. E. Do M.-K. Q. Daneshvar N. Suzuki T. Dort J. Mizunoya W. (2017). The Role of semaphorin3A in Myogenic Regeneration and the Formation of Functional Neuromuscular Junctions on New Fibres. Biol. Rev. 92 (3), 1389–1405. 10.1111/brv.12286 27296513

[B3] Anderson J. E. (2000). A Role for Nitric Oxide in Muscle Repair: Nitric Oxide-Mediated Activation of Muscle Satellite Cells. MBoC 11 (5), 1859–1874. 10.1091/mbc.11.5.1859 10793157 PMC14889

[B4] Anderson J. E. (2006). The Satellite Cell as a Companion in Skeletal Muscle Plasticity: Currency, Conveyance, Clue, Connector and Colander. J. Exp. Biol. 209 (Pt 12), 2276–2292. 10.1242/jeb.02088 16731804

[B5] Baron P. Scarpini E. Meola G. Santilli I. Conti G. Pleasure D. (1994). Expression of the Low-Affinity NGF Receptor during Human Muscle Development, Regeneration, and in Tissue Culture. Muscle Nerve 17 (3), 276–284. 10.1002/mus.880170304 8107704

[B6] Buller A. J. Eccles J. C. Eccles R. M. (1960). Interactions between Motoneurones and Muscles in Respect of the Characteristic Speeds of Their Responses. J. physiology 150 (2), 417–439. 10.1113/jphysiol.1960.sp006395 PMC1363172 13805874

[B7] Carlson B. M. Faulkner J. A. (1983). The Regeneration of Skeletal Muscle Fibers Following Injury. Med. Sci. Sports Exerc. 15 (3), 187–198. 10.1249/00005768-198315030-00003 6353126

[B8] Chargé S. B. P. Rudnicki M. A. (2004). Cellular and Molecular Regulation of Muscle Regeneration. Physiol. Rev. 84 (1), 209–238. 10.1152/physrev.00019.2003 14715915

[B9] Chen W. Datzkiw D. Rudnicki M. A. (2020). Satellite Cells in Ageing: Use it or Lose it. Open Biol. 10 (5), 200048. 10.1098/rsob.200048 32428419 PMC7276531

[B10] Chiappalupi S. Sorci G. Vukasinovic A. Salvadori L. Sagheddu R. Coletti D. (2020). Targeting RAGE Prevents Muscle Wasting and Prolongs Survival in Cancer Cachexia. J. Cachexia, Sarcopenia Muscle 11 (4), 929–946. 10.1002/jcsm.12561 32159297 PMC7432590

[B11] Cooper R. N. Tajbakhsh S. Mouly V. Cossu G. Buckingham M. Butler-Browne G. S. (1999). *In Vivo* satellite Cell Activation via Myf5 and MyoD in Regenerating Mouse Skeletal Muscle. JCell Sci. 112 (Pt 17), 2895–2901. 10.1242/jcs.112.17.2895 10444384

[B12] Cornelison D. D. W. Wold B. J. (1997). Single-cell Analysis of Regulatory Gene Expression in Quiescent and Activated Mouse Skeletal Muscle Satellite Cells. Dev. Biol. 191 (2), 270–283. 10.1006/dbio.1997.8721 9398440

[B13] Daneshvar N. Tatsumi R. Peeler J. Anderson J. E. (2020). Premature Satellite Cell Activation before Injury Accelerates Myogenesis and Disrupts Neuromuscular Junction Maturation in Regenerating Muscle. Am. J. Physiology-Cell Physiology 319 (1), C116–C128. 10.1152/ajpcell.00121.2020 32374678

[B14] Darabid H. Perez-Gonzalez A. P. Robitaille R. (2014). Neuromuscular Synaptogenesis: Coordinating Partners with Multiple Functions. Nat. Rev. Neurosci. 15 (11), 703–718. 10.1038/nrn3821 25493308

[B15] Deponti D. Buono R. Catanzaro G. De Palma C. Longhi R. Meneveri R. (2009). The Low-Affinity Receptor for Neurotrophins p75NTR Plays a Key Role for Satellite Cell Function in Muscle Repair Acting via RhoA. MBoC 20 (16), 3620–3627. 10.1091/mbc.e09-01-0012 19553472 PMC2777922

[B16] Domingues-Faria C. Vasson M.-P. Goncalves-Mendes N. Boirie Y. Walrand S. (2016). Skeletal Muscle Regeneration and Impact of Aging and Nutrition. Ageing Res. Rev. 26, 22–36. 10.1016/j.arr.2015.12.004 26690801

[B17] Donato R. Sorci G. Riuzzi F. Arcuri C. Bianchi R. Brozzi F. (2009). S100B's Double Life: Intracellular Regulator and Extracellular Signal. Biochimica Biophysica Acta (BBA) - Mol. Cell. Res. 1793 (6), 1008–1022. 10.1016/j.bbamcr.2008.11.009 19110011

[B18] Donato R. R. Cannon B. Sorci G. Riuzzi F. Hsu K. J. Weber D. (2013). Functions of S100 Proteins. Curr. Mol. Med. 13 (1), 24–57. 10.2174/156652413804486214 22834835 PMC3707951

[B19] Egan B. Zierath J. R. (2013). Exercise Metabolism and the Molecular Regulation of Skeletal Muscle Adaptation. Cell. metab. 17 (2), 162–184. 10.1016/j.cmet.2012.12.012 23395166

[B20] Englund D. A. Murach K. A. Dungan C. M. Figueiredo V. C. Vechetti I. J. Jr. Dupont-Versteegden E. E. (2020). Depletion of Resident Muscle Stem Cells Negatively Impacts Running Volume, Physical Function, and Muscle Fiber Hypertrophy in Response to Lifelong Physical Activity. Am. J. Physiology-Cell Physiology 318 (6), C1178–C1188. 10.1152/ajpcell.00090.2020 PMC7311742 32320286

[B21] Englund D. A. Figueiredo V. C. Dungan C. M. Murach K. A. Peck B. D. Petrosino J. M. (2021). Satellite Cell Depletion Disrupts Transcriptional Coordination and Muscle Adaptation to Exercise. Funct. (Oxf) 2 (1), zqaa033. 10.1093/function/zqaa033 PMC8179974 34109314

[B22] Feige P. Brun C. E. Ritso M. Rudnicki M. A. (2018). Orienting Muscle Stem Cells for Regeneration in Homeostasis, Aging, and Disease. Cell. stem Cell. 23 (5), 653–664. 10.1016/j.stem.2018.10.006 30388423 PMC6262894

[B23] Fisher T. (2019). Introduction to Celleste™ Imaging Analysis Software. [Available from:.file:///C:/Users/Owner/Desktop/Celleste_Training_Guide_UG.pdf.

[B24] Grumbles R. M. Almeida V. W. Casella G. T. B. Wood P. M. Hemstapat K. Thomas C. K. (2012). Motoneuron Replacement for Reinnervation of Skeletal Muscle in Adult Rats. J. Neuropathol. Exp. Neurol. 71 (10), 921–930. 10.1097/nen.0b013e31826cf69a 22964786 PMC3760019

[B25] Hassan S. M. Jennekens F. G. I. Veldman H. Oestreicher B. A. (1994). GAP-43 and p75NGFR Immunoreactivity in Presynaptic Cells Following Neuromuscular Blockade by Botulinum Toxin in Rat. J. Neurocytol. 23 (6), 354–363. 10.1007/bf01666525 8089707

[B26] Ip F. C. F. Cheung J. Ip N. Y. (2001). The Expression Profiles of Neurotrophins and Their Receptors in Rat and Chicken Tissues during Development. Neurosci. Lett. 301 (2), 107–110. 10.1016/s0304-3940(01)01603-2 11248434

[B27] Kang H. Lichtman J. W. (2013). Motor Axon Regeneration and Muscle Reinnervation in Young Adult and Aged Animals. J. Neurosci. 33 (50), 19480–19491. 10.1523/jneurosci.4067-13.2013 24336714 PMC6618761

[B28] H. Kang J. Lubischer C. Newman W. Thompson P. Krieg (Editors) (2000). *In Vivo* Imaging of Terminal Schwann Cells Using GFP Expressing Transgenic Mice (Washington, DC: Society for Neuroscience Abstracts).

[B29] Kang H. Tian L. Mikesh M. Lichtman J. W. Thompson W. J. (2014). Terminal Schwann Cells Participate in Neuromuscular Synapse Remodeling during Reinnervation Following Nerve Injury. J. Neurosci. 34 (18), 6323–6333. 10.1523/jneurosci.4673-13.2014 24790203 PMC4004816

[B30] Kirsch M. Campos Friz M. Vougioukas V. I. Hofmann H.-D. (2009). Wallerian Degeneration and Axonal Regeneration after Sciatic Nerve Crush Are Altered in ICAM-1-Deficient Mice. Cell. Tissue Res. 338 (1), 19–28. 10.1007/s00441-009-0837-3 19657676

[B31] Küst B. M. Copray J. C. Brouwer N. Troost D. Boddeke H. W. (2002). Elevated Levels of Neurotrophins in Human Biceps Brachii Tissue of Amyotrophic Lateral Sclerosis. Exp. Neurol. 177 (2), 419–427. 10.1006/exnr.2002.8011 12429188

[B32] Lee H. K. Shin Y. K. Jung J. Seo S.-Y. Baek S.-Y. Park H. T. (2009). Proteasome Inhibition Suppresses Schwann Cell Dedifferentiationin Vitroandin Vivo. Glia 57 (16), 1825–1834. 10.1002/glia.20894 19455715

[B33] Leiter J. R. S. Upadhaya R. Anderson J. E. (2012). Nitric Oxide and Voluntary Exercise Together Promote Quadriceps Hypertrophy and Increase Vascular Density in Female 18-Mo-Old Mice. Am. J. Physiology-Cell Physiology 302 (9), C1306–C1315. 10.1152/ajpcell.00305.2011 22322971

[B34] Lemke G. Chao M. (1988). Axons Regulate Schwann Cell Expression of the Major Myelin and NGF Receptor Genes. Development 102 (3), 499–504. 10.1242/dev.102.3.499 2846259

[B35] Love F. M. Thompson W. J. (1998). Schwann Cells Proliferate at Rat Neuromuscular Junctions during Development and Regeneration. J. Neurosci. 18 (22), 9376–9385. 10.1523/jneurosci.18-22-09376.1998 9801376 PMC6792891

[B36] McMahan U. J. Edgington D. R. Kuffler D. P. (1980). Factors that Influence Regeneration of the Neuromuscular Junction. J. Exp. Biol. 89 (1), 31–42. 10.1242/jeb.89.1.31 7009777

[B37] Megeney L. A. Kablar B. Garrett K. Anderson J. E. Rudnicki M. A. (1996). MyoD Is Required for Myogenic Stem Cell Function in Adult Skeletal Muscle. Genes Dev. 10 (10), 1173–1183. 10.1101/gad.10.10.1173 8675005

[B38] Menorca R. M. G. Fussell T. S. Elfar J. C. (2013). Nerve Physiology. Hand Clin. 29 (3), 317–330. 10.1016/j.hcl.2013.04.002 23895713 PMC4408553

[B39] Mizunoya W. Okamoto S. Miyahara H. Akahoshi M. Suzuki T. Do M.-K. Q. (2017). Fast-to-slow Shift of Muscle Fiber-type Composition by Dietary Apple Polyphenols in Rats: Impact of the Low-Dose Supplementation. Anim. Sci. J. 88 (3), 489–499. 10.1111/asj.12655 27417667

[B40] Mousavi K. Jasmin B. J. (2006). BDNF Is Expressed in Skeletal Muscle Satellite Cells and Inhibits Myogenic Differentiation. J. Neurosci. 26 (21), 5739–5749. 10.1523/jneurosci.5398-05.2006 16723531 PMC6675269

[B41] Mukaka M. M. (2012). Statistics Corner: A Guide to Appropriate Use of Correlation Coefficient in Medical Research. Malawi Med. J. 24 (3), 69–71. https://www-ncbi-nlm-nih-gov.uml.idm.oclc.org/pmc/articles/PMC3576830/ 23638278 PMC3576830

[B42] Navarro D. Foxcroft D. (2018). Learning Statistics with Jamovi: A Tutorial for Psychology Students and Other Beginners. Version 0.70.

[B43] Panzer J. A. Song Y. Balice-Gordon R. J. (2006). *In Vivo* imaging of Preferential Motor Axon Outgrowth to and Synaptogenesis at Prepatterned Acetylcholine Receptor Clusters in Embryonic Zebrafish Skeletal Muscle. J. Neurosci. 26 (3), 934–947. 10.1523/jneurosci.3656-05.2006 16421313 PMC6675385

[B44] Pasterkamp R. J. Verhaagen J. (2006). Semaphorins in Axon Regeneration: Developmental Guidance Molecules Gone Wrong? Phil. Trans. R. Soc. B 361 (1473), 1499–1511. 10.1098/rstb.2006.1892 16939971 PMC1664670

[B45] Qaisar R. Bhaskaran S. Van Remmen H. (2016). Muscle Fiber Type Diversification during Exercise and Regeneration. Free Radic. Biol. Med. 98, 56–67. 10.1016/j.freeradbiomed.2016.03.025 27032709

[B46] Reddy L. V. Koirala S. Sugiura Y. Herrera A. A. Ko C.-P. (2003). Glial Cells Maintain Synaptic Structure and Function and Promote Development of the Neuromuscular Junction *In Vivo* . Neuron 40 (3), 563–580. 10.1016/s0896-6273(03)00682-2 14642280

[B47] Reynolds M. L. Woolf C. J. (1992). Terminal Schwann Cells Elaborate Extensive Processes Following Denervation of the Motor Endplate. J. Neurocytol. 21 (1), 50–66. 10.1007/bf01206897 1346630

[B48] Riuzzi F. Beccafico S. Sorci G. Donato R. (2016). S100B Protein in Skeletal Muscle Regeneration: Regulation of Myoblast and Macrophage Functions. Eur. J. Transl. Myol. 26 (1), 5830. 10.4081/ejtm.2016.5830 27054019 PMC4821221

[B49] Riuzzi F. Beccafico S. Sagheddu R. Chiappalupi S. Giambanco I. Bereshchenko O. (2017). Levels of S100B Protein Drive the Reparative Process in Acute Muscle Injury and Muscular Dystrophy. Sci. Rep. 7 (1), 12537. 10.1038/s41598-017-12880-9 28970581 PMC5624904

[B50] Roberson M. D. Toews A. D. Bouldin T. W. Weaver J. Goines N. D. Morell P. (1995). NGFR-mRNA Expression in Sciatic Nerve: a Sensitive Indicator of Early Stages of Axonopathy. Mol. Brain Res. 28 (2), 231–238. 10.1016/0169-328x(94)00211-v 7723622

[B51] Roux P. Barker P. A. (2002). Neurotrophin Signaling through the P75 Neurotrophin Receptor. Prog. Neurobiol. 67 (3), 203–233. 10.1016/s0301-0082(02)00016-3 12169297

[B52] Sabourin L. A. Girgis-Gabardo A. Seale P. Asakura A. Rudnicki M. A. (1999). Reduced Differentiation Potential of Primary MyoD−/− Myogenic Cells Derived from Adult Skeletal Muscle. J. Cell. Biol. 144 (4), 631–643. 10.1083/jcb.144.4.631 10037786 PMC2132931

[B53] Sagheddu R. Chiappalupi S. Salvadori L. Riuzzi F. Donato R. Sorci G. (2018). Targeting RAGE as a Potential Therapeutic Approach to Duchenne Muscular Dystrophy. Hum. Mol. Genet. 27 (21), 3734–3746. 10.1093/hmg/ddy288 30085099

[B54] Saika T. Senba E. Noguchi K. Sato M. Yoshida S. Kubo T. (1991). Effects of Nerve Crush and Transection on mRNA Levels for Nerve Growth Factor Receptor in the Rat Facial Motoneurons. Brain Res. Mol. Brain Res. 9 (1-2), 157–160. 10.1016/0169-328x(91)90142-k 1850072

[B55] Sanes J. R. Lichtman J. W. (1999). Development of the Vertebrate Neuromuscular Junction. Annu. Rev. Neurosci. 22, 389–442. 10.1146/annurev.neuro.22.1.389 10202544

[B56] Schmalbruch H. Lewis D. M. (2000). Dynamics of Nuclei of Muscle Fibers and Connective Tissue Cells in Normal and Denervated Rat Muscles. Muscle Nerve 23 (4), 617–626. 10.1002/(sici)1097-4598(200004)23:4<617:aid-mus22>3.0.co;2-y 10716774

[B57] Schor N. F. (2005). The P75 Neurotrophin Receptor in Human Development and Disease. Prog. Neurobiol. 77 (3), 201–214. 10.1016/j.pneurobio.2005.10.006 16297524

[B58] Seidl K. Erck C. Buchberger A. (1998). Evidence for the Participation of Nerve Growth Factor and its Low-Affinity Receptor (p75NTR) in the Regulation of the Myogenic Program. J. Cell. Physiol. 176 (1), 10–21. 10.1002/(sici)1097-4652(199807)176:1<10:aid-jcp2>3.0.co;2-b 9618140

[B59] Son Y.-J. Thompson W. J. (1995). Schwann Cell Processes Guide Regeneration of Peripheral Axons. Neuron 14 (1), 125–132. 10.1016/0896-6273(95)90246-5 7826630

[B60] Sorci G. Riuzzi F. Agneletti A. L. Marchetti C. Donato R. (2004). S100B Causes Apoptosis in a Myoblast Cell Line in a RAGE-independent Manner. J. Cell. Physiol. 199 (2), 274–283. 10.1002/jcp.10462 15040010

[B61] Sugiura Y. Lin W. (2011). Neuron-glia Interactions: the Roles of Schwann Cells in Neuromuscular Synapse Formation and Function. Biosci. Rep. 31 (5), 295–302. 10.1042/bsr20100107 21517783 PMC4573580

[B62] Suzuki T. Do M.-K. Q. Sato Y. Ojima K. Hara M. Mizunoya W. (2013). Comparative Analysis of Semaphorin 3A in Soleus and EDL Muscle Satellite Cells *In Vitro* toward Understanding its Role in Modulating Myogenin Expression. Int. J. Biochem. Cell. Biol. 45 (2), 476–482. 10.1016/j.biocel.2012.10.003 23085379

[B63] Tajbakhsh S. (2003). Stem Cells to Tissue: Molecular, Cellular and Anatomical Heterogeneity in Skeletal Muscle. Curr. Opin. Genet. Dev. 13 (4), 413–422. 10.1016/s0959-437x(03)00090-x 12888016

[B64] Tatsumi R. Allen R. E. (2008). Mechano-biology of Resident Myogenic Stem Cells: Molecular Mechanism of Stretch-Induced Activation of Satellite Cells. Anim. Sci. J. 79, 279–290. 10.1111/j.1740-0929.2008.00528.x 20163667

[B65] Tatsumi R. Sankoda Y. Anderson J. E. Sato Y. Mizunoya W. Shimizu N. (2009). Possible Implication of Satellite Cells in Regenerative Motoneuritogenesis: HGF Upregulates Neural Chemorepellent Sema3A during Myogenic Differentiation. Am. J. Physiology-Cell Physiology 297 (2), C238–C252. 10.1152/ajpcell.00161.2009 19515904

[B66] Tatsumi R. Suzuki T. Do M.-K. Q. Ohya Y. Anderson J. E. Shibata A. (2017). Slow-Myofiber Commitment by Semaphorin 3A Secreted from Myogenic Stem Cells. Stem Cells 35 (7), 1815–1834. 10.1002/stem.2639 28480592

[B67] Toma J. Pareek S. Barker P. Mathew T. Murphy R. Acheson A. (1992). Spatiotemporal Increases in Epidermal Growth Factor Receptors Following Peripheral Nerve Injury. J. Neurosci. 12 (7), 2504–2515. 10.1523/jneurosci.12-07-02504.1992 1377231 PMC6575829

[B68] Toti P. Villanova M. Vatti R. Schuerfeld K. Stumpo M. Barbagli L. (2003). Nerve Growth Factor Expression in Human Dystrophic Muscles. Muscle Nerve 27 (3), 370–373. 10.1002/mus.10332 12635125

[B69] Wosczyna M. N. Rando T. A. (2018). A Muscle Stem Cell Support Group: Coordinated Cellular Responses in Muscle Regeneration. Dev. Cell. 46 (2), 135–143. 10.1016/j.devcel.2018.06.018 30016618 PMC6075730

[B70] Yano H. Chao M. V. (2000).Neurotrophin Receptor Structure and Interactions, Pharmacochem. Libr., 31, 253–260. 10.1016/s0165-7208(00)80026-4 10812966

